# Real-Time Deflection Monitoring for Milling of a Thin-Walled Workpiece by Using PVDF Thin-Film Sensors with a Cantilevered Beam as a Case Study

**DOI:** 10.3390/s16091470

**Published:** 2016-09-10

**Authors:** Ming Luo, Dongsheng Liu, Huan Luo

**Affiliations:** Key Laboratory of Contemporary Design and Integrated Manufacturing Technology, Ministry of Education, Northwestern Polytechnical University, Xi’an 710072, China; 2014200870@mail.nwpu.edu.cn (D.L.); louris@mail.nwpu.edu.cn (H.L.)

**Keywords:** real-time monitoring, workpiece deflection, vibration, PVDF sensor, thin-walled workpiece, thin-film sensor

## Abstract

Thin-walled workpieces, such as aero-engine blisks and casings, are usually made of hard-to-cut materials. The wall thickness is very small and it is easy to deflect during milling process under dynamic cutting forces, leading to inaccurate workpiece dimensions and poor surface integrity. To understand the workpiece deflection behavior in a machining process, a new real-time nonintrusive method for deflection monitoring is presented, and a detailed analysis of workpiece deflection for different machining stages of the whole machining process is discussed. The thin-film polyvinylidene fluoride (PVDF) sensor is attached to the non-machining surface of the workpiece to copy the deflection excited by the dynamic cutting force. The relationship between the input deflection and the output voltage of the monitoring system is calibrated by testing. Monitored workpiece deflection results show that the workpiece experiences obvious vibration during the cutter entering the workpiece stage, and vibration during the machining process can be easily tracked by monitoring the deflection of the workpiece. During the cutter exiting the workpiece stage, the workpiece experiences forced vibration firstly, and free vibration exists until the amplitude reduces to zero after the cutter exits the workpiece. Machining results confirmed the suitability of the deflection monitoring system for machining thin-walled workpieces with the application of PVDF sensors.

## 1. Introduction

Thin-walled workpieces, such as aero-engine blisks and casings, are usually made of hard-to-cut materials (titanium alloy or Ni-based alloy). The wall thickness is very small and it is easy to deflect during the milling process. For example, the leading and trailing edge of the compressor blade is less than 0.5 mm. Additionally, the cutting force is usually very large in the milling of these hard-to-cut materials [1]. Thus, the cutting force will force the workpiece to deflect during the milling process. However, due to the complex structure of the thin-walled workpiece, it is difficult and time-consuming to predict the workpiece deflection by the means of analytical prediction or finite element (FE) simulation. On the other hand, since the milling process is highly interrupted and the cutter enters and exits the workpiece frequently, the deflection of the workpiece is more complex than the static loading case. Deflection of the workpiece during the milling process will lead to inaccurate workpiece dimensions and poor surface integrity, such as a wavy surface. However, the machining accuracy and surface integrity is important for thin-walled workpieces to ensure the service life and working performance. Understanding the basic physics is the key issue for the machining process control. In a milling process, material removal has a significant influence on the dynamic properties of the workpiece, which will further influence the dynamic response of the workpiece during the machining process. Due to the structural complexity of the workpiece, and the nonlinearity of machining system, numerical simulation is very difficult and time-consuming. Therefore, real-time monitoring of the dynamic response opens the door for the investigation of complex machining processes [2].

Polyvinylidene fluoride (PVDF) is a kind of organic polymer piezoelectric material and is the piezoelectric polymer material with the most potential [3,4]. PVDF-based sensors have been frequently used in industry for energy harvesting [5], vibration monitoring and control [6], pressure monitoring [7,8], dynamic force measurement [9], and machining process monitoring [10,11]. Some previous studies have been focused on the application of PVDF sensors for deformation or deflection monitoring, as well as dynamic sensing. Yi and Liang [12] designed a PVDF-based deformation sensor for tire rubber deformation and insect locomotion studies. It was later embedded on the inner tread surface of the tire to get critical information for understanding and estimating wheel-ground interactions [13]. Ye et al. [14] developed a PVDF-based sensor to detect the change of maximum displacement from an ionic exchange polymer metal composite actuator in real-time. Zeng et al. [15] developed a sensor that was a hybrid of carbon black and PVDF for use in in situ acquisition of dynamic elastic disturbances of low frequency vibrations. Ma et al. [11] mounted the PVDF sensor on the milling cutter to measure the cutting forces in a machining process. The measured data can be further used for on-line chatter detection in milling processes [10]. However, little research has demonstrated that the PVDF sensors have ever been used to monitor the workpiece deflection in the milling process. 

Machining process monitoring with sensors is one of the most important research topics in manufacturing; the monitoring system can be used for machining condition monitoring and for machining process optimization to improve the machining quality and efficiency [16]. Among the developed monitoring systems, tool conditions and machining vibrations are the two main foci. For tool condition monitoring, tool wear, or breakage [17,18], tool deflection during the machining process has been studied by many researchers. Based on the monitored signals, machining parameters can be optimized to improve the machining quality or efficiency [19]. For tool deflection monitoring in the milling process, it has been developed and integrated into the spindle [20]. For workpiece deflection under dynamic cutting forces, it is one of the most fundamental physical phenomena, which will affect the workpiece quality as well as its service performance [21]. A commonly used method for workpiece deflection identification is to use in-process force to calculate workpiece deflection. However, due to the uncertainties and difficulties in cutting force measurement, calculated workpiece deflection is not accurate. Moreover, due to the existence of nonlinear phenomena, such as loss contact between the cutter and the workpiece, it is difficult to get the exact workpiece deflection. To capture real-time deflection during the machining process, some sensors, such as thickness probes and acceleration sensors, have been used in the machining process [2]. However, the above-mentioned method is more suitable for large-scale workpiece monitoring, such as aircraft frame structures. Moreover, the use of these measuring techniques will affect, or even change, the machining process; thus, the measured data is not the same in cases without the measurement device. 

To understand the workpiece deflection behavior in the machining process, it is natural to call for the development of a nonintrusive monitoring method during the machining process, and this is the objective of the work reported in this paper. We will present a new nonintrusive method for deflection monitoring and analysis for the whole machining process of thin-walled workpiece. Firstly, we described the theoretical background of the PVDF deflection and charge generation. Next, a nonintrusive monitoring system is presented, and the deflection (input)—voltage (output) relationship for the thin-film sensor is established through physical calibration. Finally, workpiece deflection and characteristics under different machining stages are discussed. The presented method provides insight into the mechanism of dynamic deflection of the workpiece. The contribution of this study are: (1) a nonintrusive monitoring method is developed for real-time workpiece deflection monitoring in the milling of thin-walled workpieces; and (2) the monitored workpiece signals reveal that for different machining stages, the workpiece deflection or vibration modes are different. This helps to give a better understanding of the thin-walled workpiece deflection and vibration behavior during the milling process. Furthermore, the monitored real-time deflection data can be used for machining process improvement of thin-walled workpieces. 

This paper is structured as follows: the next section gives the theoretical background of PVDF deflection; Section 3 provides the nonintrusive monitoring system by taking a titanium alloy cantilevered beam as a case study; Section 4 then discusses the workpiece deflection under different cutting stages; and, finally, the conclusion in Section 5. 

## 2. Theoretical Background of PVDF Deflection

**Figure 1:**
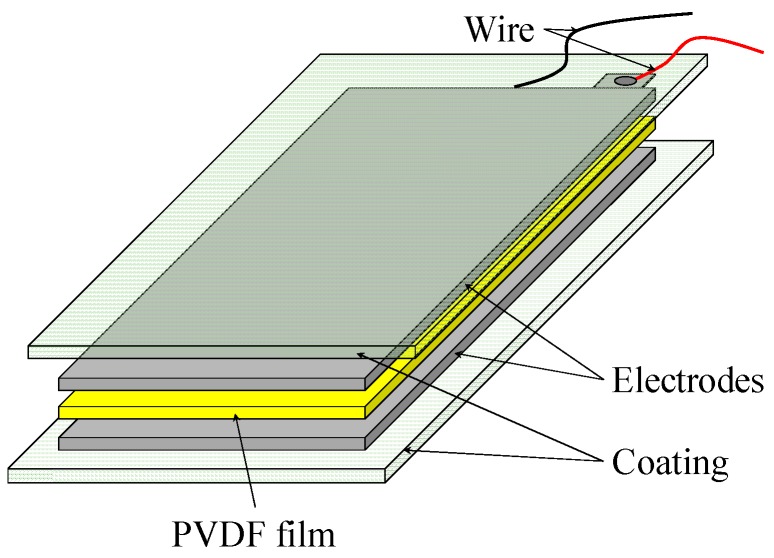
Schematic configuration of the PVDF sensor.

In this study, PVDF thin-film sensors are used to monitor the deflection of a thin-walled workpiece. As shown in Figure 1, the PVDF sensor consists of a PVDF film with electrodes on both sides, and the electrodes are connected with wires to output the generated charge. There are coatings on both sides of the sensor to protect the sensor from damage [22]. The total thickness of the sensor can vary from 10 μm to hundreds of micrometers according to its application cases. The thin-film sensors will generate charge once it deflects or sustains normal pressure. The thickness of the PVDF film used in this study is 28 μm, the piezo strain constant $d_{31}=23\times 10^{-12}$ PC/N, the relative dielectric constant is 12, and the volume resistivity is $10^{13}$ Ohm meters.

The charge is then amplified by a charge amplifier circuit [23], converted into digital signals, and sent to the PC. The PVDF thin-film sensor can work within a wide frequency range with high sensitivity, it has good repeatability and reliability. In addition to the good repeatability, the PVDF sensor is sensitive to the dynamic input, the generated charge will be released under the static state. In other words, the PVDF thin film can be pre-deflected and it will not affect the dynamic measurement results. Therefore, it is suitable for workpiece deflection monitoring.

### 2.1. Charge Calculation under Deflection

**Figure 2:**
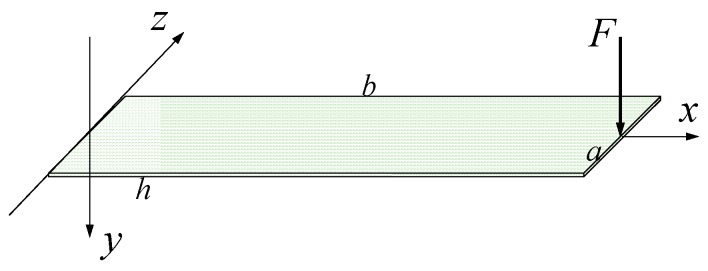
The cantilever-based PVDF sensor.

In this section, we first consider the output model for the charge generated by the deflection of the PVDF thin-film. Consider a cantilever-based PVDF thin-film as shown in Figure 2, the width, length and thickness of the PVDF film are a, b, and h, respectively. As shown in Figure 2, when a force is applied on the tip of the free end of the film, it will deflect and generate charge. For the PVDF film, we ignore the shear stress and assume the charge is generated by the piezoelectric effect along the *x*-direction since the film is very thin. Thus the accumulated electric charge can be calculated as [24].

(1) $$Q_{en}=\int_{A}d_{31}\sigma_{1}dA$$

where $d_{31}$ is the PVDF piezoelectric constant between the 3 and 1 directions, $\sigma_{1}$ is the normal stress across the film section, and A is the electrode area of the PVDF surface [22]. Since only small deflection exists, we assume that the electric charge is mainly generated by the deflection of the PVDF film. The stress $\sigma_{1}$ of the PVDF film is calculated as:

(2) $$\sigma_{1}=\frac{My}{I_{z}}$$

Then Equation (1) can be written as:

(3) $$Q_{en}=\int_{A}d_{31}\sigma_{1}dA=\frac{3d_{31}b^{2}}{2h^{2}}F$$

Then the output charge is transformed into voltage. The output voltage can be described as:

(4) $$V=\frac{Q}{C_{f}}$$

where $C_{f}$ is the capacitance of the negative feedback capacitor of the charge amplifier circuit. According to the above derivation and Equations (1)–(4), once the PVDF thin-film deflects, voltage will be output. Therefore, we can establish the relationship between the workpiece (or thin-film) deflection and the output signals. 

### 2.2. Mechanical Model of the PVDF Sensor

The PVDF sensor is attached at the free end of the workpiece. Therefore, the deflection of the workpiece will be transferred to the PVDF thin-film during the milling process. The deflection function of a cantilever beam can be expressed as [25]:

(5) $$\frac{1}{\rho \left(x\right)}=\frac{M\left(x\right)}{EI_{z}}=\pm \frac{\frac{d^{2}y}{dx^{2}}}{{\left(1+{\left(\frac{dy}{dx}\right)}^{2}\right)}^{\frac{3}{2}}}$$

where ρ is the curvature radius of neutral layer, E is the Young’s module of the PVDF film, M is the bending moment, $I_{z}$ is the moment of inertia of z-axis cross section. Since $\frac{dy}{dx}=\theta$ is usually small, then the approximated differential equation of deflection curve is:

(6) $$\frac{d^{2}y}{dx^{2}}=\frac{M\left(x\right)}{EI_{z}}$$

By using integration, the deflection function for the thin PVDF film is derived as:

(7) $$y=\frac{1}{EI_{z}}\int \left(\int Mdx\right)dx+Cx+D$$

where C and D are integral constants. 

For the cantilever thin-film, the boundary condition for fixed end is:

$$\{\begin{array}{c} \theta_{\mathrm{fix}}=0,\;x=0\\ y_{\mathrm{fix}}=0,\;x=0 \end{array}$$

By submitting the above boundary condition into Equation (7), we can get:

(8) $$C=-\frac{1}{2}Fb^{2}$$

(9) $$D=\frac{1}{6}Fb^{3}$$

Then we can get the deflection of the film under external applied force F:

(10) $$y=\frac{F}{EI_{z}}\left(\frac{1}{6}{\left(x-b\right)}^{2}-\frac{1}{2}b^{2}x+\frac{1}{6}b^{3}\right)$$

Here, we use $I_{z}=\frac{1}{12}ah^{3}$ for the film’s rectangular cross-section area. At the free end, where x=b, the maximum deflection is:

(11) $$y_{max}=\frac{4b^{3}}{Eah^{3}}F$$

Since the real-time cutting position is always shifting, the deflection on the cutting position can be calculated according to Equation (10). Then the deflection transferred to the free end will be recorded by the PVDF sensor.

## 3. Real-Time Workpiece Deflection Monitoring with PVDF Thin-Film

**Figure 3:**
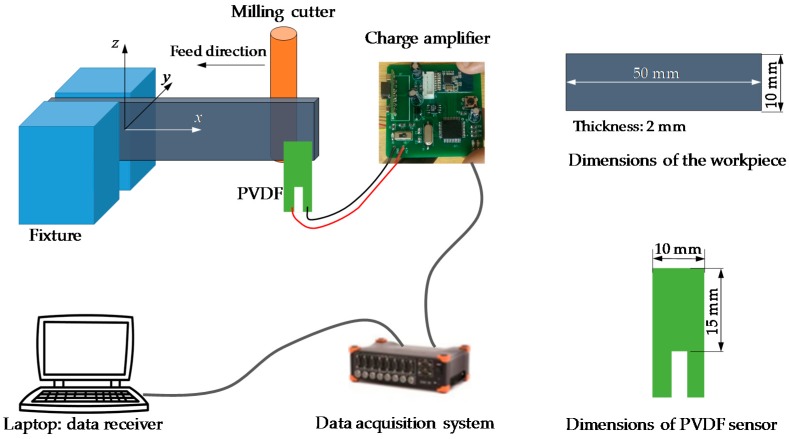
Flow of the real-time monitoring of workpiece deflection.

For the purpose of a better testing of the nonintrusive monitoring method, we will take a titanium alloy cantilevered beam as a case study. The material of the thin-walled workpiece is Ti_6_Al_4_V, and its dimensions are 2.0 mm in thickness, 10.0 mm in height, and 50.0 mm in length. The overall flow chart is shown in Figure 3. The presented thin-walled workpiece deflection monitoring solution has four main components: the PVDF thin-film sensor, the charge amplifier circuit, the data acquisition system, and the data receiver. Once the PVDF sensor is forced to deflect due to the deflection of the workpiece, the generated charge will be amplified and sent to the data acquisition system for analysis. During the machining process, only one side of the workpiece is machined. Then the thin-film PVDF sensor is attached to the other side which will not be machined. Since the sensor is highly flexible, it will not affect the workpiece-fixture system and it will copy the deflection of the cantilevered beam at its free end. Comparing with the sensor’s upper cut-off frequency (above 10 MHz), the vibration frequency of the workpiece due to the dynamic cutting force is much smaller, thus, it is good enough to monitor the deflection of the workpiece. The overlapping between the sensor and the workpiece is 1.0 mm to keep the sensor contact with the workpiece during the milling process, the sensor is pre-deflected since the workpiece will deflect in both positive and negative directions of the y-axis due to vibration. The overlapping should be small to get the simple bending motion of the sensor. 

**Figure 4:**
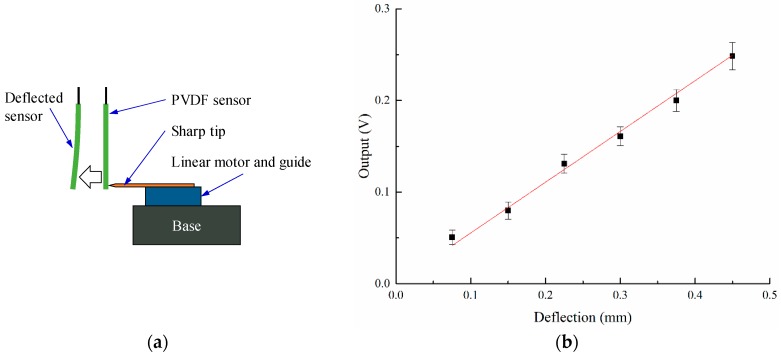
Calibration of the deflection-voltage relationship of the monitoring system. (**a**) Calibration setup; and (**b**) calibration results.

The input of the monitoring system is the deflection of the PVDF sensor, and the output is the voltage; we have to establish the relationship between the input (deflection) and the output (voltage). Thus, we can get the deflection amplitude of the workpiece during the machining process. The schematic setup for calibration is shown in the Figure 4a. A linear motor carrying a sharp tip can move along the linear guide, the accurate displacement of the motor can be controlled. When the tip touches the PVDF sensor and keeps moving forward, the PVDF sensor will generate charge under deflection. For different displacements, the generated charge is different. The test for the same displacement was repeated five times. The calibrated result is shown in Figure 4b.

Once we get signals for the whole machining process, we can identify the exact positions where unacceptable workpiece deflection or vibration happens. After that, modified machining parameters can be identified for these positions to keep the machining quality of following workpieces within an acceptable range. Furthermore, once the online real-time adjustment systems are available, we can adjust the machining parameters (feedrate, spindle speed) to avoid large deflection and vibrations for the current workpiece to keep the machining quality within an acceptable range.

## 4. Milling Results and Discussions

**Figure 5:**
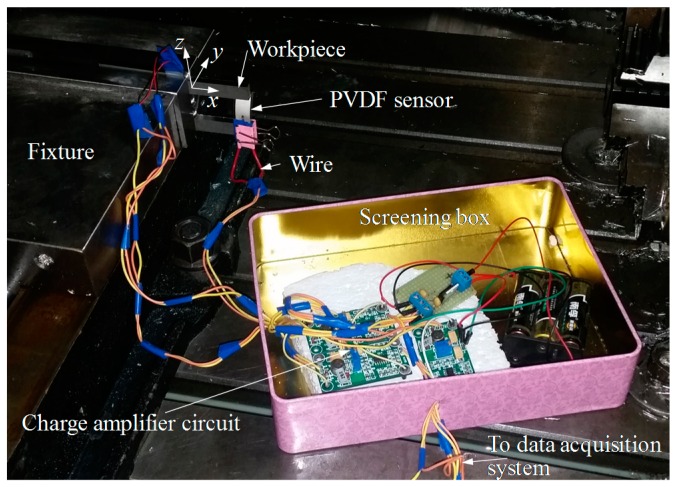
Experimental setup.

**Figure 6:**
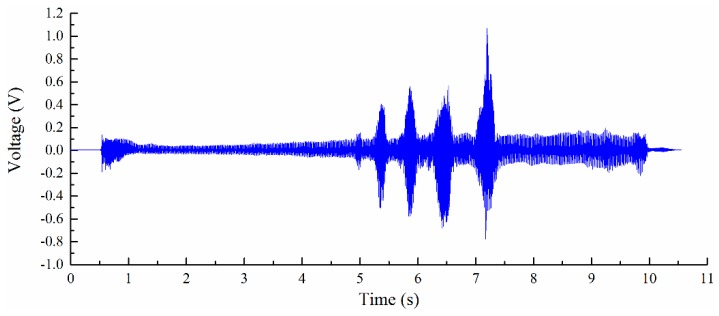
Output signals of the real-time monitoring system.

The experimental setup is shown in Figure 5; the cutter is a flat end milling cutter with a 10 mm diameter and four teeth. The spindle speed is 3000 rpm; thus, the time for one cutter revolution is 0.02 s and the time for one tooth is 0.005 s. The feedrate is set to 360 mm/min. The initial thickness of the workpiece is 2.0 mm, it is expected to be machined to 1.0 mm, the radial depth of cut is 1.0 mm and the axial depth of cut is 10.0 mm, and overhang of the workpiece is 50.0 mm. The deflection monitoring point locates at the free end of the cantilever beam (workpiece), and real-time deflection at the free end was monitored with the PVDF thin-film sensor. Real-time output signals of the monitoring system are shown in Figure 6.

**Figure 7:**
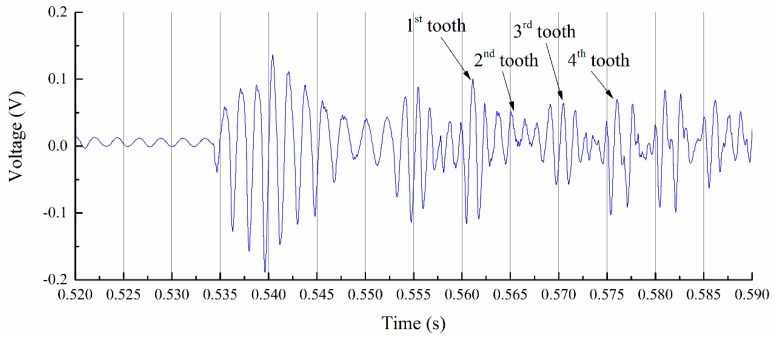
Initial cutting stage.

**Figure 8:**
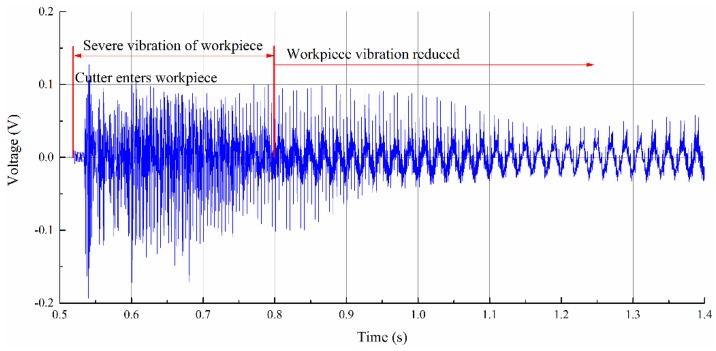
Transition from the vibration stage to the stable cutting stage.

The monitored cutter’s entering of the workpiece stage is shown in Figure 7. From the results, we can identify each tooth clearly. Monitored deflection results show that the workpiece experienced vibration after the cutter teeth entered the workpiece. During the entering process, the cutting process provides dynamic input force and leads to the forced vibration of the thin-walled workpiece. After the cutter teeth exited the workpiece, the workpiece experienced a short time of free vibration. Before the vibration amplitude reduced to zero, the next tooth entered the workpiece. Monitored signals of the workpiece show that the cutter’s entering process of the workpiece is not stable; it lasts less than 1 s and goes to a stable cutting process, as shown in Figure 8.

**Figure 9:**
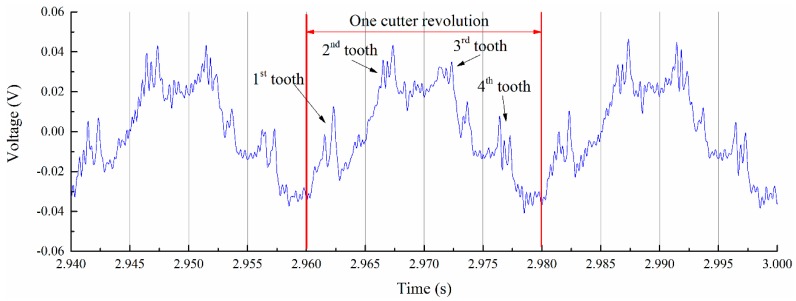
Workpiece deflection in the normal cutting stage.

For the stable cutting stage, the workpiece deflection is very complex, but the deflection amplitude is very small, as shown in Figure 9. For each cutter revolution period, the workpiece deflections are almost the same. From the results we can also see that the workpiece did not experience free vibration during stable cutting situation.

**Figure 10:**
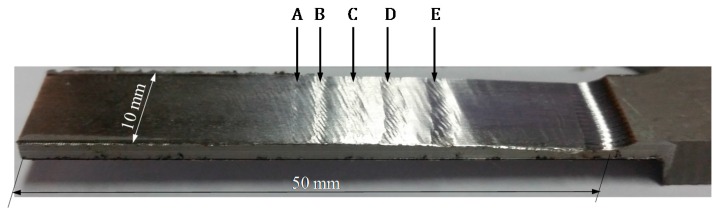
Machined workpiece with vibration marks.

**Figure 11:**
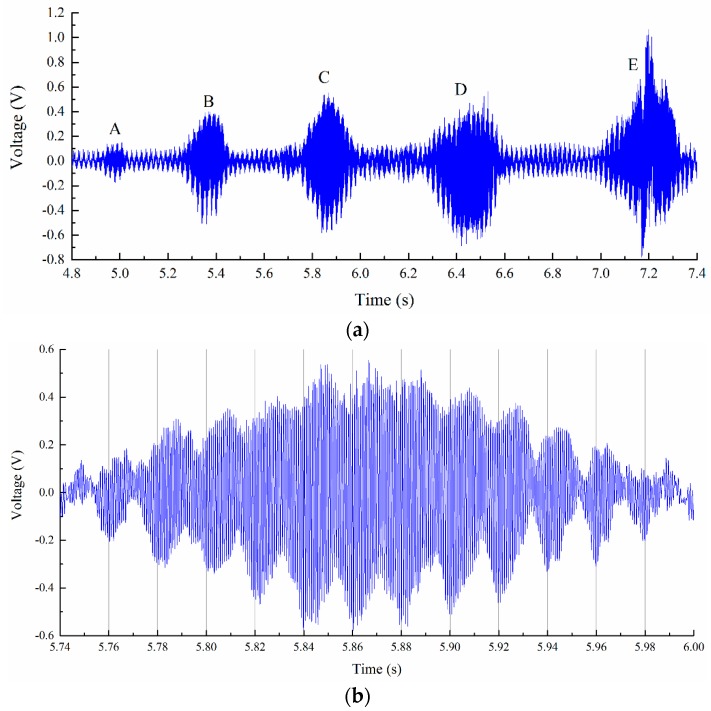
Vibration stage. (**a**) Workpiece vibration; and (**b**) enlarged view of workpiece vibration signals at position C.

**Figure 12:**
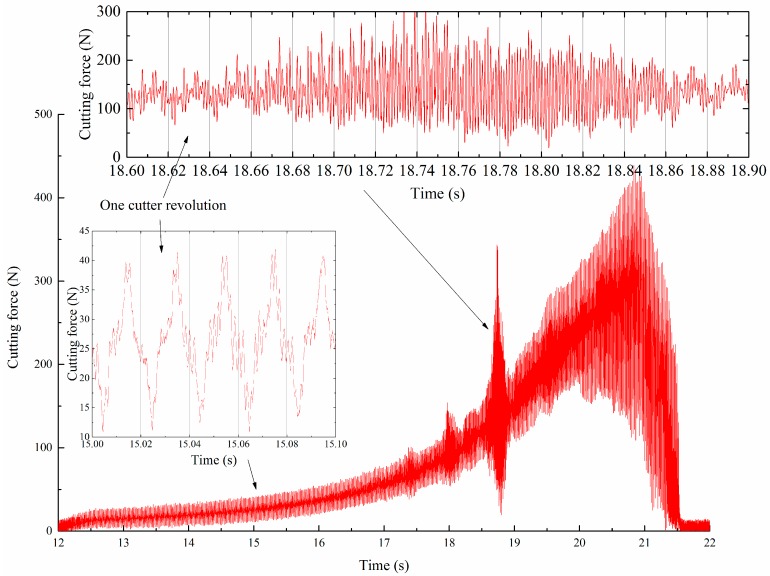
Measured cutting force during the machining of a thin-walled workpiece.

For the cutting period from 4.8 s to 7.4 s, workpiece vibrations were observed from the monitored signals, the vibration marks are shown in Figure 10. The corresponding monitored signals are shown in Figure 11. From the results we can see that the workpiece experienced severe vibration under the cutting forces, as shown in Figure 11b. Monitored signals clearly show that there were five positions where vibration happened, and all the vibrations left marks on the workpiece surface. For comparison, cutting forces during the milling process were also recorded by the Kistler 9123C dynamometer mounted on the machine tool spindle, the recorded cutting forces are shown in Figure 12. We can see that during the stable cutting process, the force profile is clear and each cutter rotation period can be easily identified. While machining vibration happened, the cutting force profile changed dramatically. Comparing with cutting force signals, the deflections monitored with PVDF sensors are more remarkable. The results show that the PVDF thin-film is suitable for real-time machining vibration monitoring. 

**Figure 13:**
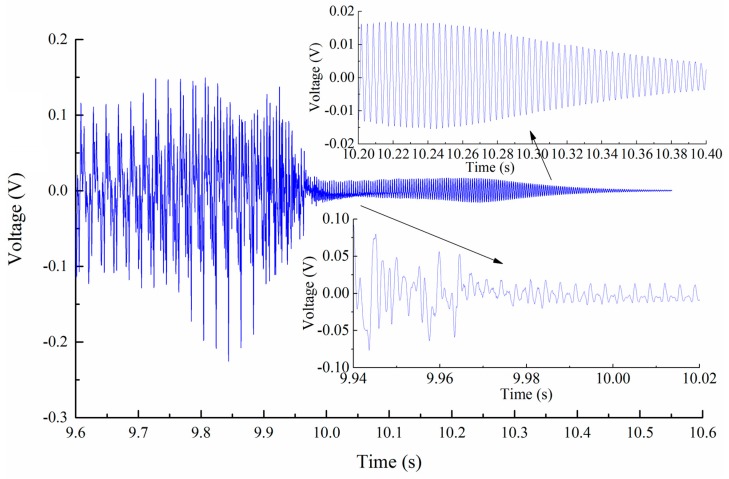
Workpiece deflection during the cutter exiting stage.

For the cutter exiting workpiece stage, the workpiece experienced obvious vibration. As shown in Figure 13, the workpiece vibration was disturbed by the cutter tooth entering actions during rotation before the cutter exited the workpiece. After the cutter exited the workpiece, the thin-walled workpiece experienced the standard free vibration until the amplitude reduced to zero.

## 5. Conclusions

A new real-time nonintrusive method for deflection monitoring is presented, and detailed analysis of workpiece deflection for different machining stages of the whole machining process of thin-walled workpieces is discussed in this paper. Machining results confirm the suitability of a deflection monitoring system for machining thin-walled workpieces with the application of PVDF sensors. Monitored signals of the PVDF sensor reveal that, for different machining stages, the workpiece deflection or vibration modes are different. This helps to give a better understanding of the thin-walled workpiece vibration behavior during the process. Regarding potential future research, more PVDF sensors can be embedded into the fixture to obtain more complex deflections of completed structures, thus providing insight and understanding of workpiece behavior during the machining process for high-value-added components, such as aero-engine casings. More compact circuits for charge amplifiers and signal transmission will be developed. Moreover, position-dependent analysis and optimization of the machining process will also be carried out.

## References

[1] Luo M., Wang J., Wu B., Zhang D. (2016). Effects of cutting parameters on tool insert wear in end milling of titanium alloy Ti6Al4V. Chin. J. Mech. Eng..

[2] Li Y., Liu C., Gao J.X., Shen W. (2015). An integrated feature-based dynamic control system for on-line machining, inspection and monitoring. Integr. Comput.-Aided Eng..

[3] Dung V.C., Sasaki E. (2016). Numerical simulation of output response of PVDF sensor attached on a cantilever beam subjected to impact loading. Sensors.

[4] Asif K., Zafar A., Heung Soo K., Il-Kwon O. (2016). Piezoelectric thin films: An integrated review of transducers and energy harvesting. Smart Mater. Struct..

[5] Yoon S.-J., Arakawa K., Uchino M. (2015). Development of an energy harvesting damper using PVDF film. Int. J. Energy Res..

[6] Ma C.C., Chuang K.C., Pan S.Y. (2011). Polyvinylidene fluoride film sensors in collocated feedback structural control: Application for suppressing impact-induced disturbances. IEEE Trans. Ultrason. Ferroelectr. Freq. Control.

[7] Shirinov A.V., Schomburg W.K. (2008). Pressure sensor from a PVDF film. Sens. Actuators A Phys..

[8] Kimoto A., Shimada S. (2013). A proposal of new multifunctional pressure sensor based on PVDF films. IEEE Trans. Instrum. Meas..

[9] Yu P., Liu W., Gu C., Cheng X., Fu X. (2016). Flexible piezoelectric tactile sensor array for dynamic three-axis force measurement. Sensors.

[10] Ma L., Melkote S.N., Castle J.B. (2013). A model-based computationally efficient method for on-line detection of chatter in milling. J. Manuf. Sci. Eng..

[11] Ma L., Melkote S.N., Morehouse J.B., Castle J.B., Fonda J.W., Johnson M.A. (2012). Thin-film PVDF sensor-based monitoring of cutting forces in peripheral end milling. J. Dyn. Syst. Meas. Control.

[12] Yi J., Liang H. (2008). A PVDF-based deformation and motion sensor: Modeling and experiments. IEEE Sens. J..

[13] Yi J. (2008). A piezo-sensor-based “Smart Tire” system for mobile robots and vehicles. IEEE/ASME Trans. Mechatron..

[14] Ye X., Zhu L., Guo S., Li Y. Research on PVDF displacement sensor used on IPMC. Proceedings of the 2009 International Conference on Mechatronics and Automation.

[15] Zeng Z., Liu M., Xu H., Liu W., Liao Y., Jin H., Zhou L., Zhang Z., Su Z. (2016). A coatable, light-weight, fast-response nanocomposite sensor for the in situ acquisition of dynamic elastic disturbance: From structural vibration to ultrasonic waves. Smart Mater. Struct..

[16] Stavropoulos P., Chantzis D., Doukas C., Papacharalampopoulos A., Chryssolouris G. (2013). Monitoring and control of manufacturing processes: A review. Procedia CIRP.

[17] Nouri M., Fussell B.K., Ziniti B.L., Linder E. (2015). Real-time tool wear monitoring in milling using a cutting condition independent method. Int. J. Mach. Tools Manuf..

[18] Hou Y., Zhang D., Wu B., Luo M. (2015). Milling force modeling of worn tool and tool flank wear recognition in end milling. IEEE/ASME Trans. Mechatron..

[19] Tapoglou N., Mehnen J., Vlachou A., Doukas M., Milas N., Mourtzis D. (2015). Cloud-based platform for optimal machining parameter selection based on function blocks and real-time monitoring. J. Manuf. Sci. Eng..

[20] Möhring H.C., Litwinski K.M., Gümmer O. (2010). Process monitoring with sensory machine tool components. CIRP Ann. Manuf. Technol..

[21] Kersting P., Biermann D. (2014). Modeling techniques for simulating workpiece deflections in NC milling. CIRP J. Manuf. Sci. Technol..

[22] Measurement Specialties Inc. (2002). Piezo Film Sensors Technical Manual.

[23] Marinov A.S., Stanchev O.P., Bekov E.B. Application of charge amplifiers with Polyvinylidene Fluoride materials. Proceedings of the 2014 37th International Convention on Information and Communication Technology, Electronics and Microelectronics (MIPRO).

[24] Standards Committee of IEEE Ultrasonics (1987). Ferroelectrics, and Frequency Control Society.

[25] Beer F.P., Russell Johnston E., Dewolf J.T., Mazurek D.F. (2012). Mechanics of Materials.

